# Patterns and Predictors of Medication Change after Discharge from Hospital: An Observational Study in Older Adults with Neurological Disorders

**DOI:** 10.3390/jcm11030563

**Published:** 2022-01-23

**Authors:** Anna Schwarzkopf, Aline Schönenberg, Tino Prell

**Affiliations:** 1 Department of Neurology, Jena University Hospital, 07747 Jena, Germany; anna.schwarzkopf@med.uni-jena.de (A.S.); tino.prell@med.uni-jena.de (T.P.); 2 Department of Geriatrics, Halle University Hospital, 06120 Halle, Germany

**Keywords:** hospital discharge, medication change, polypharmacy, potentially inappropriate medication, elderly, adherence, Parkinson’s disease

## Abstract

Background: Medication is often changed after inpatient treatment, which affects the course of the disease, health behavior and adherence. Thus, it is important to understand patterns of medication changes after discharge from hospital. Methods: Inpatients at the Department of Neurology received a comprehensive assessment during their stay, including adherence, depression, cognition, health and sociodemographic variables. A month after being discharged, patients were contacted to enquire about post-discharge medication changes. Results: 910 older adults aged 70 ± 8.6 years participated, of which 204 (22.4%) reported medication changes. The majority of changes were initiated by physicians (*n* = 112, 56.3%) and only 25 (12.6%) patients reported adjusting medication themselves. Reasons for medication changes differed between patients and doctors (*p* < 0.001), with side effects or missing effects cited frequently. Sociodemographic and patient-related factors did not significantly predict medication changes. Conclusion: Patients reported less post-discharge medication changes than expected, and contrary to previous literature on nonadherence, only a fraction of those changes were performed by patients themselves. Socioeconomic and clinical parameters regarding personality, mood and cognition were poorly associated with post-discharge medication changes. Instead, individual health-related factors play a role, with patient factors only indirectly influencing physicians’ decisions.

## 1. Introduction

The treatment of chronic disorders commonly includes the long-term use of pharmacotherapy. This is particularly true for older adults who are often expected to adhere to complex drug regimens [1]. In addition to this initial complexity, medication is frequently adjusted after hospital discharge. A previous study reported that only 21% of geriatric patients remain on their initial discharge prescriptions [2], and another group of researchers cite a medication change prevalence of over 50% post-discharge [3]. Especially general practitioners (GP) extensively adjust medication after discharge [2]. Some authors reported that more than half of the discharge prescriptions among 726 geriatric participants were classified as potentially inappropriate [4], whereas a different study found a reduction in inappropriate prescriptions during the stay at a specialized geriatric care hospital compared to admission medication [5].

These frequent changes in medication impact medication knowledge and adherence to medication and thus also health outcomes and hospital readmissions [6]. Adherence is used as a term to describe how well a person follows the recommendations from healthcare providers, such as changing the diet and exercise, taking part in therapies, and taking prescribed medication [7]. However, many older adults either do not want to or cannot take medications as agreed upon [8]. This nonadherence to medication increases the chances for adverse drug events and readmissions to hospitals, higher costs, lower quality of life and general poorer health outcomes [7,9,10,11]. Factors contributing to nonadherence are numerous and can be divided into patient-, physician- and healthcare-related factors. On the patient side, several factors leading to nonadherence have been identified, such as depressive mood, cognition and healthcare beliefs [9,12]. On the other hand, complexity and frequent medication changes contribute to poorer knowledge about and adherence to medication regimes [6,9,13]. In general, nonadherence may be intentional (when the patient purposefully decides not to follow the recommended treatment, e.g., due to beliefs or perceived risks) or unintentional (when the patient cannot follow the recommendation, e.g., due to lack of understanding or forgetting). Especially the motivators behind intentional nonadherence are crucial to understand, as this represents a willful, conscious decision to interfere with medication recommendations that can be targeted in interventions [13].

As inpatient hospital care provides an ideal moment for optimizing medication [3], it is crucial to understand how medication regimes change during the transition from inpatient to outpatient care. While some medication is planned to be short-term and needs to be adjusted post-discharge, e.g., when medication was prescribed due to acute events, it is crucial to discern the patterns of changes made erroneously. The transition from inpatient to outpatient care is accompanied by a myriad of changes, adjustments and barriers and can often lead to adverse effects due to miscommunication, lack of understanding or inappropriate changes [14]. During most hospital stays, a new medication plan is put together [15], and preventing unplanned medication changes after discharge is of high relevance to ensure continued health and prevent readmission. Combining the reports of high levels of both post-discharge medication changes [2,3] and nonadherence in older adults [14,16,17] poses the question of which quota of these changes are induced by patients themselves. To the best of our knowledge, this distinction between different initiators of medication changes has not been made before, but it is crucial to disentangle different agents and their motivations for medication changes to improve health outcomes and intervene with appropriate measures if needed. Therefore, we aimed to explore the exact nature of these medication changes to understand how and why patients modify their medication after discharge. Based on the cited literature, we expected a high level of post-discharge medication changes, with a significant portion of those changes initiated by patients themselves. The goal of the current analysis is therefore to first describe the nature of medication changes after discharge from hospital in older adults, and to understand the factors predicting these medication changes. For this purpose, we interviewed patients during their inpatient stay about their medication intake and collected comprehensive patient-related data. Four weeks after discharge, we contacted the patients via telephone to enquire about potential medication changes.

## 2. Materials and Methods

### 2.1. Setting and Participantss

This cross-sectional study was registered in the German Clinical Trials Register DRKS00016774 (registered 19 February 2019) and the study protocol was published in advance (Prell, 2019). The study was approved by the local ethics committee (approval number 5290-10/17) of Jena University Hospital. All patients provided written informed consent.

As a summary, from February 2019 to March 2020, patients 60 years of age or older with neurological disorders received a comprehensive geriatric assessment during their stay in the Department of Neurology at the Jena University Hospital, Jena, Germany. The data used for this analysis is freely available [18].

This paper reports analyses of the longitudinal dataset with the first follow-up investigation. The following assessments were used for this analysis: age, gender, main neurological diagnosis, medication regime at admission and discharge, marital status (single/divorced/widowed or married), living condition (alone, not alone), level of education (high, middle, low), number of medications per day, medical diagnoses. Additionally, the following assessments were made: depression (Beck Depression inventory, BDI II) [16,17], personality (Big Five Inventory, BFI) [19], health-care climate (HCCQ) [20], health-related quality of life (HRQoL) using SF-36 [21], adherence (Stendal Adherence to Medication Score, SAMS) [22], timed-up-and-go-test (TuG) [23], and Montreal Cognitive Assessment (MoCa) [24].

The SF-36 is a general health-related QoL questionnaire to assess with the following 8 different domains: problems regarding both physical and social activity due to health, limitations in daily life due to physical or emotional problems, pain, mental health, vitality, and general health perception. Each domain is summarized as the weighted sum of the respective items, with lower scores indicating less disability. A physical and mental compound score as well subscores can be calculated [21]. The Stendal Adherence to Medication Scale (SAMS) is a questionnaire with 18 items summarized in a cumulative adherence score, with 0 indicating complete adherence and 72 complete nonadherence [22]. Different facets of adherence are included, namely modification of medication, lack of knowledge and forgetting to take medication [22,25]. For the follow-up assessment approximately one month after discharge, patients were contacted via telephone and the following questions were asked: “How would you rate your current health in comparison to the time before your hospital stay—better, about the same, or worse?”, “Were there any changes to your medication since your discharge from hospital?”, “if yes, which ones?”, “if yes, by whom were these changes made?”, and “if yes, why were the changes made?”.

### 2.2. Statistical Analysis

In the first step, the cohort was summarized with descriptive statistics. Normal distribution was assessed with Shapiro–Wilk test. The different groups of patients were compared using the Student’s *t*-test, Mann–Whitney U test or Chi-squared test with Bonferroni correction for multiple comparisons where appropriate. Binominal logistic regression with backward selection was used to determine the association between changes of medication after discharge and the variables BDI score, number of medication and Sf-36 subscales Physical Functioning and Health Change. Linearity was tested assessed using the Box–Tidwell [26]. Bonferroni correction was applied to all terms in the model [27]. All variables were found to follow a linear relationship. Correlations between predictor variables were low (r < 0.70), indicating that multicollinearity was not a confounding factor in the analysis. Goodness-of-fit was assessed using the Hosmer–Lemeshow test. We generally apply a significance level of 0.05 and 2-sided tests.

## 3. Results

### 3.1. Clinical and Demographical Characteristics

The baseline assessment included 910 older adults (389 female and 521 male) aged 70 ± 8.6 years (see Supplementary Table S1 for a detailed cohort description). Most patients were married, pensioned, lived with family members and had a high education level; most predominant diagnoses were movement disorders (33.3%), followed by cerebrovascular (25.6%) and neuromuscular (18.5%) disorders. The majority of patients had no (*n* = 468, 51.4%) or minimal depression (*n* = 187, 20.5%), a mild, moderate or severe depression was observed in 139 (15.3%), 61 (6.7%) or 27 (3%) patients, respectively. Cognition was normal in 466 (51.2%; MoCA ≥ 23) and cognitive deficits were present in 370 (40.7%, MoCA < 23) persons (74 missing data). For follow-up one month after discharge, 712 persons (78.2%) were interviewed; 192 (21.1%) could not be reached, 6 (0.7%) died.

### 3.2. Patterns of Medication Changes after Discharge

**Figure 1:**
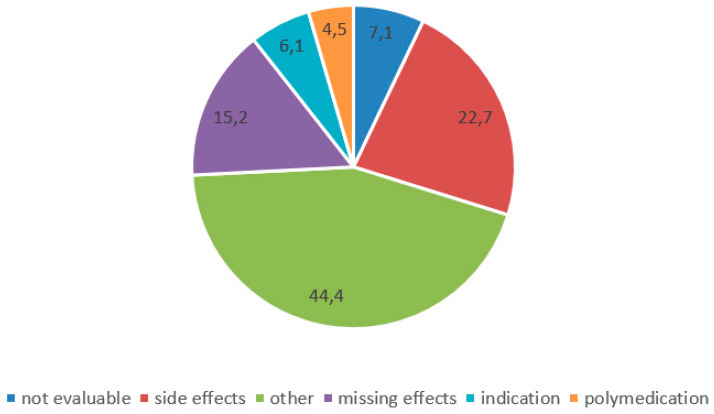
Reasons for changes of medication after discharge.

**Figure 2:**
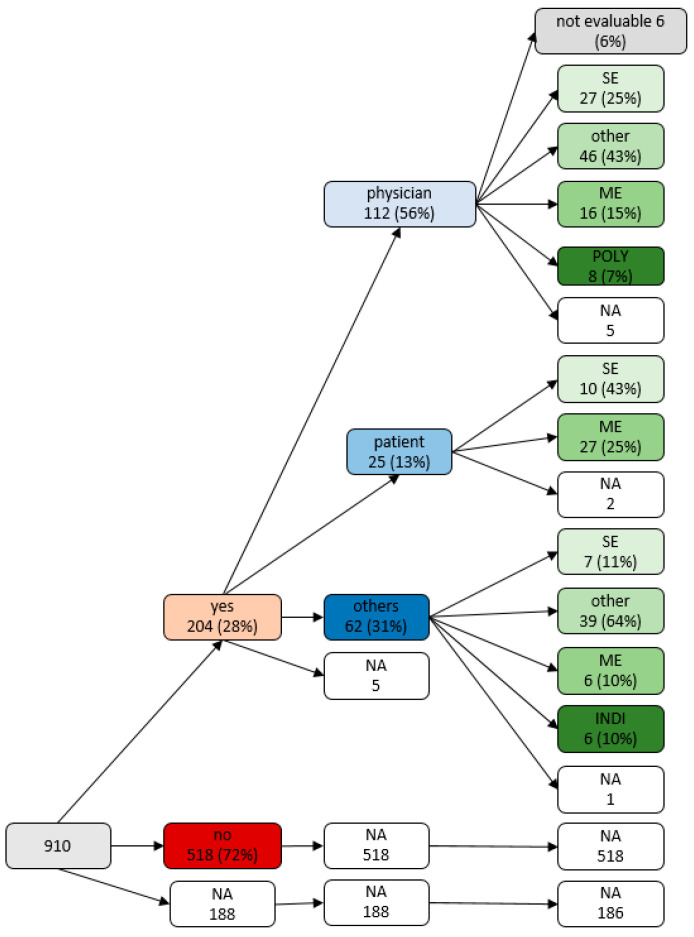
Variable tree: initiator with their different reasons for changes of medication after discharge, note: NA = not available, INDI = new indication, ME = missing effects, SE = side, effects, POLY = polymedication.

Changes of medication after discharge were reported by 204 (22.4%) patients. These medication changes were mainly initiated by physicians (*n* = 112, 56.3%, by GP 74, by Neurologist 34, by other hospitals/rehabilitation centers 4); only a minority of patients reported initiating medication changes themselves (*n* = 25, 12.6%) (*n* = 62, 31.2% by others). Reasons for medication changes were side effects, missing effects and miscellaneous reasons (individual reasons such as planned operations, specific allergies, or unavailability of certain medications) (Figure 1). We summarized the initiators of changes and the corresponding reasons for change in a variable tree in Figure 2.

The composition of initiators and reasons for medication changes is further illustrated in the alluvial diagram in Supplementary Figure S1. The type of changes differed between the indicator of those changes (χ^2^ = 979.6, *p* < 0.001), with changes made by patients themselves mainly related to side effects and missing effect, whereas changes made by physicians were due to side effects or a collection of individual, miscellaneous reasons (see Supplementary Figure S2).

### 3.3. Predictors of General Medication Changes after Discharge

In the univariate group comparison, changes of medication were more common in people with depression and self-reported nonadherence (Table 1). In terms of HRQoL, people with medication changes reported better physical functioning and less worsening of health before assessment (domain health change) (Table 1). In the corresponding binomial logistic regression, neither the SAMS nor the BDI were able to significantly predict medication change after discharge (model χ^2^ = 3.48, *p* = 0.175).

Although in the binominal logistic regression the combination of physical function and health change significantly predicted medication changes after discharge, the explained variance was low, as shown by Nagelkerke’s R^2^ = 0.019 (Table 2).

### 3.4. Predictors of Patient-Initiated Medication Changes after Discharge

Given that we hypothesized that different mechanisms may underlie the changes made by the patients (i.e., changes were made without consulting a doctor) and changes made by physicians, we performed additional analyses with respect to the initiator of medication changes, i.e., patient vs. physician. In the univariate analysis patients who initiated changes themselves were more frequently female (Table 3).

Both groups did not differ in terms of overall adherence assessed by the total SAMS (Table 3) nor on SAMS item level (Supplementary Table S2).

In the binominal logistic regression only female gender (OR = 2.91, CI [0.890, 9.53], *p* = 0.077), and higher age (OR = 1.057, CI [0.987, 1.132], *p* = 0.114) were associated with patient-initiated medication changes after discharge (χ^2^ = 6.06, *p* = 0.048, Nagelkerke’s R^2^ = 0.10) (Supplementary Table S3).

Medication changes due to side effects were not related to any of the assessed clinical parameters and occurred independent of age, gender, diagnosis, depression or adherence. Medication changes due to missing effect were more frequent in people with movement disorders (e.g., Parkinson’s disease) (Table 4).

## 4. Discussion

This study examined patterns and predictors of medication changes in older adults after discharge from hospital. One month after discharge, the medication of 22.4% of the patients was changed, which is lower than previously reported in the literature [2,3]. Additionally, contrary to our hypotheses based on high levels of nonadherence in older patients [14,16,17], only a small fraction of the patients reported performing medication changes themselves, with most of the changes predominantly issued by outpatient physicians. This is at odds with previous literature reporting that 40% of patients were nonadherent to at least one medication after discharge [28]. Likewise, Mansur et al. (2008) showed that 36.7% of patients reported changes in their medication one month after discharge, with 30% of patients being nonadherent to at least one medication [29]. Different prevalence of medication changes may partially be explained by different follow-up time intervals. Additionally, a differentiation must be made between acute and planned hospital stays, as Mansur, Weiss, Hoffman, Gruenewald and Beloosesky [29] recruited their patients from the acute geriatric ward. As shown in our analyses, patient-related indicators including sociodemographic and personal parameters such as HRQoL, adherence, mood and cognition were not or only poorly associated with changes of medication after discharge, which mirrors the findings by Mansur, Weiss, Hoffman, Gruenewald and Beloosesky [29]. We initially hypothesized that nonadherence could be a predictor of medication change as previously demonstrated for a smaller cohort of people with Parkinson’s disease [30]. However, this could not be replicated in this cohort of older adults with mixed disorders. These results indicate that, although the literature often focuses on nonadherent or “difficult” patients [31,32,33,34], the majority of patients do not willfully interfere with their medication plan after discharge from hospital. This interpretation is supported by the finding that adherence rates did not differ between patients who initiated changes themselves and patients where the physicians initiated changes. These findings display how it is not adherence per se that contributes to adjustments of medication. On the SAMS item-level we also did not observe contributions of different reasons of nonadherence (i.e., missing knowledge about medication, intentional modification of medication, forgetting) to medication changes after discharge. However, we cannot exclude that that adherence-related patient factors may influence medication changes at a later timepoint after discharge.

It seems that health-related factors such as side effects or lack of effects as well as individual health-related factors play a role for prompt medication changes. Especially the high rate of “other” reasons for post-discharge medication changes in our study again highlights the role of individual, specific reasons that cannot be generalized. However, since the present analysis was focused on predicting patient-related factors that lead to medication changes after discharge, individual health-related factors were not assessed and only a cautious indication can be made on the basis of the small but significant prediction of physical functioning and health change on medication changes.

We found that changes due to missing effects are more common in Parkinson´s disease than in the other disorder groups. This is probably due to the fact that, in the case of Parkinson’s disease, medication is frequently adjusted during the inpatient stay and certain expectations of a prompt improvement in motor function are associated with this [35]. If this improvement does not occur, people with Parkinson´s disease seem to be more willing to adjust medication on their own. Understandably, however, this explanation cannot be transferred to other diseases, such as vascular diseases, where there is no immediate benefit of medication. In general, adherence patterns may vary depending on the underlying diagnosis [36,37,38], as different diagnoses may come with varying levels of chronicity, restrictions and burden [9]. Thus, predictors and barriers for adherence can differ between patient groups [39].

Overall, it can only be cautiously stated on the basis of the calculated models that the probability of patient-initiated medication changes appears to be higher in older women. However, the interpretability of this results is reduced by the small group of patients that initiated medication changes themselves, thus the collected patient-related parameters such as mood, cognition or adherence could not fully capture the reasons for medication changes. Patient-related factors may indirectly indicate medication changes by influencing the physician, e.g., if a patient reports depressive mood changes leading to the physician administering a dosage reduction, but overall, our results suggest that medication change seems to be both health-related and physician-indicated.

This study has several limitations. First, the results are restricted to hospitalized neurogeriatric patients. Given that we were mainly interested in personal factors, we used self-report instruments to detect nonadherence and medication change after discharge. We did not compare the list of prescribed medications with the actual medications taken. Although this is a common and valid approach, it does not allow a statement about the real medication possession ratio or correctness of drug intake. Self-reports are known to be prone to systematic and unsystematic biases [40], especially regarding social desirability and recall bias. However, self-reported adherence measurements can still provide reliable information on adherence levels and can detect clinically relevant nonadherence [41]. All questionnaires used are valid and commonly used in the clinical literature. Although we collected a large amount of clinical data, it is not possible to capture all relevant factors, and due to our hypothesis-driven approach the current data cannot explain all factors pertaining to medication changes. Thus, further studies are needed to understand the individual health-related factors leading to the physician-initiated medication changes and their effects on health outcomes. In addition, to fully understand the effect of patient-related factors, it would be of interest to analyze their indirect effects on the physician’s decisions in greater detail.

As a general conclusion from our data, we can say that medication changes shortly after hospital discharge are less frequent than expected in this cohort of older adults with neurological disorders. Furthermore, they are predominantly performed by physicians for individual health-related reasons rather than patient nonadherence. However, as not all medication changes are clinically appropriate and may lead to adverse health events [42], it is crucial to optimize communication between patients, hospitals, and physicians to assess all relevant individual factors and achieve the best possible health outcomes.

## Figures and Tables

**Table 1:** Group comparison: cross table for dependent medication change after discharge.

	*n*	Medication Change: Yes (*n* = 204)	Medication Change: No (*n* = 518)	*p*
		% (Frequency)	% (Frequency)	
sex	910			0.99 ^1^
male		58.3% (119/204)	58.3% (302/518)	
female		41.7% (85/204)	41.7% (216/518)	
Education level	896			0.42 ^1^
High		36.2% (72/199)	34.8% (179/513)	
Middle		31.6% (63/199)	36.6% (188/513)	
Low		32.2% (64/199)	28.6% (146/513)	
Diagnosis group	910			0.71 ^1^
movement disorder		34.3% (70/204)	35.5% (184/518)	
cerebrovascular disorder		27.9% (57/204)	23.1% (120/518)	
Epilepsy		4.4% (9/204)	4.4% (23/518)	
neuromuscular		18.1% (37/204)	18.9% (98/518)	
Others		15.2 (31/204)	17.9 (93/518)	
BFI	843			0.55 ^1^
Neuroticism		15.3% (28/182)	10.7% (51/476)	
Openness		13.7% (25/182)	16.1% (77/476)	
Conscientiousness		42.3% (77/182)	44.3% (211/476)	
Extraversion		20.8% (38/182)	21.0% (100/476)	
Agreeableness		7.6% (14/182)	7.7% (37/476)	
Medication change in the last 6 months: yes	844	52.7% (95/180)	44.1% (212/480)	0.05 ^1^
		**Q1 Mdn Q3**	**Q1 Mdn Q3**	***p***
Age	910	84.0 **71.0** 78.0	63.0 **70.0** 77.0	0.41 ^2^
TuG	585	8.0 **9.0** 11.0	8.0 **9.0** 11.0	0.67 ^2^
Frequency of doctor appointments (quarterly)	838	1.0 **1.0** 3.0	1.0 **1.0** 3.0	0.78 ^2^
BDI	909	6.0 **9.2** 15.0	4.0 **8.0** 14.0	<0.01 ^2^
MoCA	910	22.0 **24.0** 25.0	22.0 **23.0** 26.0	0.84 ^2^
SAMS	755	1.0 **5.0** 10.0	1.0 **4.0** 8.0	0.01 ^2^
Number of Medications	910	1.0 **5.0** 9.6	1.0 **4.0** 8.0	0.01 ^2^
HCCQ	831	5.0 **5.7** 6.4	5.1 **5.9** 6.4	0.24 ^2^
SF36				
Physical functioning	903	18.3 45.0 70.0	25.0 50.0 75.0	0.02 ^2^
Social functioning	907	50.0 75.0 100.0	50.0 75.0 100.0	0.75 ^2^
Role limitations due to physical health	874	0.0 0.0 75.0	0.0 0.0 75.0	0.91 ^2^
Role limitations due to emotional problem	875	0.0 100.0 100.0	0.0 100.0 100.0	0.28 ^2^
Emotional well-being	899	52.0 68.0 80.0	52.0 68.0 80.0	0.68 ^2^
Energy/fatigue	899	30.0 45.0 60.0	35.0 50.0 65.0	0.12 ^2^
Pain	907	22.4 44.9 77.6	32.7 55.1 77.7	0.24 ^2^
General health	896	35.0 45.0 55.0	35.0 45.0 55.0	0.35 ^2^
Health change	899	0.0 25.0 50.0	25.0 25.0 50.0	0.01 ^2^
Physical Health component score	849	24.2 33.1 41.0	26.6 33.7 43.0	0.20 ^2^
Mental Health component score	849	38.7 50.4 57.1	39.5 51.0 57.4	0.81 ^2^

^1^ Pearson. ^2^ Wilcoxon, Q1 = first quartile, Q3 = third quartile, Mdn = median. BFI = Big Five Inventory, TuG = Timed up and go, BDI = Beck Depression Inventory, MoCA = Montreal Cognitive Assessment, HCCQ = Health Care Climate Questionnaire, SAMS = Stendal Adherence to Medication Score, SF36 = Short Form 36.

**Table 2:** Binominal logistic regression: medication change after discharge (yes/no).

Step		95% Confidence Interval			Nagelkerkes R^2^			
		Exp(B)	Lower CI	Upper CI		χ^2^	df	Sig.
1	BDI	1.011	0.987	1.035	0.020	9.568	4	0.048
	Number of pills/day	0.997	0.949	1.047				
	Physical functioning	0.995	0.989	1.002				
	Health change	0.994	0.987	1.001				
	Constant	0.549						
2	BDI	1.011	0.987	1.035	0.020	9.553	3	0.023
	Physical functioning	0.995	0.989	1.002				
	Health change	0.994	0.987	1.001				
	Constant	0.539						
3	Physical functioning	0.995	0.989	1.000	0.019	8.784	2	0.012
	Health change	0.993	0.986	1.001				
	Constant	0.634						

Note: BDI = Beck Depression Inventory, health change and physical functioning measured by Short Form 36 subscales.

**Table 3:** Group comparison: cross table for initiator of medication change after discharge.

		**Physician-Initiated**				**Patient-Initiated**				***p***
		***n***	**%**			***n***	**%**			
Sex	female	41	29.9%			16	11.7%			0.012
	male	71	51.8%			9	6.6%			
Medication change	no	39	34.2%			8	7.0%			0.196
	yes	61	53.5%			6	5.3%			
Diagnosis	Movement disorder	184	25.5%			70	9.7%			0.714
	cerebrovascular disorder	120	16.6%			57	7.9%			
	epilepsy	23	3.2%			9	1.2%			
	neuromuscular	98	13.6%			37	5.1%			
	others	93	12.9%			31	4.3%			
Education level	high	45	33.6%			7	5.2%			0.469
	middle	36	26.9%			10	7.5%			
	low	28	20.9%			8	6.0%			
		**M**	**SD**	**Lower 95% CI (M)**	**Upper 95% CI (M)**	**M**	**SD**	**Lower 95% CI (M)**	**Upper 95% CI (M)**	***p***
Age		70.5	9.4	68.7	72.3	72.8	6.7	70.1	75.6	0.263
Number of pills/day		5.8	3.7	5.1	6.5	6.0	4.1	4.3	7.7	0.840
BDI		11.3	7.3	9.9	12.6	12.9	7.0	10.0	15.9	0.206
HCCQ-D		5.5	1.0	5.3	5.7	5.2	1.6	4.3	6.2	0.829
MoCA		23.7	2.4	23.2	24.1	24.1	2.2	23.2	25.0	0.313
Frequency of doctor appointments (quarterly)		2.1	3.0	1.5	2.7	1.4	0.9	0.9	1.9	0.760
SAMS total		7.0	7.2	5.7	8.4	6.4	6.2	3.8	8.9	0.621

BDI = Beck Depression Inventory, MoCA = Montreal Cognitive Assessment, HCCQ = Health Care Climate Questionnaire, SAMS = Stendal Adherence to Medication Score.

**Table 4:** Univariate comparison for different reasons of medication change (with Bonferroni correction).

		Others				Side Effects				Missing Effects				Sign.
		*n*	%			*n*	%			*n*	%			χ^2^
Sex	female	48	24.2%			21	10.6%			13	6.6%			0.655
	male	75	37.9%			24	12.1%			17	8.6%			
Diagnosis group	movement disorder	32	16.2%			19	9.6%			17	8.6%			0.082
	cerebrovascular disorder	38	19.2%			11	5.6%			7	3.5%			
	epilepsy	7	3.5%			1	0.5%			0	0.0%			
	neuromuscular	25	12.6%			6	3.0%			4	2.0%			
	others	21	10.6%			8	4.0%			2	1.0%			
Education Level	high	47	24.4%			12	6.2%			13	6.7%			0.145
	middle	39	20.2%			14	7.3%			12	6.2%			
	low	34	17.6%			18	9.3%			4	2.1%			
		**M**	**SD**	**95%CI**	**95%CI**	**M**	**SD**	**95%CI**	**95%CI**	**M**	**SD**	**95%CI**	**95%CI**	***p***
Age		69.7	8.7	68.1	71.2	71.8	8.1	69.4	74.2	71.0	9.3	67.5	74.5	<0.05
Number of pills/day		5.8	3.7	5.1	6.5	5.6	3.9	4.5	6.8	5.6	3.8	4.2	7.1	<0.05
BDI		10.5	6.7	9.3	11.7	10.8	7.4	8.6	13.0	15.2	9.3	11.6	18.7	<0.05
HCCQ-D		5.6	1.0	5.4	5.7	5.5	1.2	5.2	5.9	5.0	1.6	4.3	5.8	<0.05
MoCA		23.6	2.6	23.1	24.1	23.8	2.7	23.0	24.6	23.8	2.2	23.0	24.7	<0.05
Frequency of doctor appointments (quarterly)		2.0	2.8	1.5	2.5	2.7	2.9	1.8	3.6	1.7	1.5	1.1	2.4	<0.05
SAMS total		7.5	7.7	6.1	8.8	5.4	5.6	3.7	7.1	6.4	6.5	3.9	8.8	<0.05

Note: BDI = Beck’s Depression Inventory II, HCCQ-D = Health Care Climate Questionnaire, MoCA = Montreal Cognitive Assessment, SAMS = Stendal Adherence to Medication Score.

## Data Availability

The data used in this analysis is freely available for noncommercial scientific purposes at doi:10.5061/dryad.w3r2280qh [18].

## Supplementary Materials

The following supporting information can be downloaded at: https://www.mdpi.com/article/10.3390/jcm11030563/s1: Table S1: Clinical and demographical characteristics; Table S2: Comparison of SAMS on item level for medication changes initiated by patient or physician; Table S3: Binominal logistic regression: Medication changes initiated by patient or physician; Figure S1: Alluvial plot for reasons of medication changes; Figure S2: Scatter plot of bivariate relationship.
