# Supplementary material for: Patterns and Predictors of Medication Change after Discharge from Hospital: An Observational Study in Older Adults with Neurological Disorders
Source: J Clin Med. 2022 Jan 23;11(3):563. doi: 10.3390/jcm11030563 (PMC8836689; doi:10.3390/jcm11030563)
Supplement: Supplementary file 1 [file jcm-11-00563-s001.zip › jcm-1544386-supplementary.pdf]

Supplementary Materials

Table S1. Clinical and demographical characteristics (N = 910)

		**N**	**%**	**missing**
Sex	Female	389	42.7	0
	Male	521	57.3	
Marital	Single / widowed /divorced	277	30.8	12
	Married	621	69.2	
Living situation	Alone	204	24.1	65
	not alone	641	75.9	
Education	High	325	36.3	14
	Middle	306	34.2	
	Low	265	29.6	
Occupation	no work	756	84.0	10
	Working	144	16.0	
Diagnosis	movement disorder	303	33.3	0
	cerebrovascular disorder	233	25.6	
	Epilepsy	48	5.3	
	Neuromuscular	168	18.5	
	Others	158	17.4	
		**M**	**SD**	**missing**
Age		70.1	8.6	0
BDI II		9.8	7.6	28
HCCQ-D		5.6	1.1	79
MoCA		22.5	4.4	74
Timed Up & Go duration in seconds		10.5	4.3	68
		**M**	**95%CI**	**95%CI**
Physical functioning		47.9	45.9	50
Social functioning		71	69.2	72.8
Role limitations due to physical health		30.2	27.6	32.8
Role limitations due to emotional problem		61.9	58.9	64.9
Emotional well-being		65.1	63.9	66.4
Energy/fatigue		48.5	47.2	49.8
Pain		54.7	52.7	56.8
General health		44.4	43.3	45.5
Health change		31.7	30.1	33.3

Note: BDI = Beck's Depression Inventory II, HCCQ = Health Care Climate Questionnaire, MoCA = Montreal Cognitive Assessment, SAMS = Stendal Adherence to Medication Score

Table S2. Comparison of SAMS on item level for medication changes initiated by patient or physician

SAMS Item	**Initiator**	**N**	**M**	**SD**	**T**	**df**	**p**
1 Do you know the reason for taking your medication?	Physician	112	.50	.870	1.089	134	.278
	Patient	24	.29	.751			
2 Do you know the dosages of your medication?	Physician	112	.59	1.151	1.455	52.864	.151
	Patient	25	.32	.748			
3 Are you familiar with the timing for taking the medication?	Physician	112	.26	.720	.666	135	.506
	Patient	25	.16	.374			
4 Do you take your medication regularly?	Physician	112	.15	.506	-1.072	135	.286
	Patient	25	.28	.678			
5 Do you know the names of medications you are taking?	Physician	111	.52	.999	-.455	133	.650
	Patient	24	.63	1.013			
6 Do you forget to take your medication?	Physician	111	.77	.805	1.026	134	.307
	Patient	25	.60	.577			
7 Are you untroubled about taking the medication?	Physician	111	.85	1.336	-.391	134	.697
	Patient	25	.96	1.172			
8 Do you stop taking your medication when you feel better?	Physician	107	.36	.882	.837	130	.404
	Patient	25	.20	.577			
9 Do you stop taking your medication if you sometimes feel worse after taking the medication?	Physician	109	.35	.865	.817	132	.415
	Patient	25	.20	.577			
10 Do you take any wrong or other/unprescribed medications (such as those of your partner)?	Physician	111	.06	.432	.713	133	.477
	patient	24	.00	.000			
11 If you think you have side effects due to of the medications (such as tremors. nausea etc.). do you reduce the dose without consulting a doctor?	physician	110	.32	.823	-.085	132	.933
	patient	24	.33	.637			
12 If you think you have side effects due to of the medications (such as tremors. nausea etc.). do you not take the medication for a while. i.e. take a break?	physician	109	.32	.826	.398	131	.691
	patient	24	.25	.608			
13 If you feel you have to take too many. do you stop taking those medications you consider to be less important than the others	physician	110	.16	.567	-.024	132	.981
	patient	24	.17	.482			

without consulting your doctor?							
14 If you forget or omit your medication, do you forget it in the morning?	physician	106	.34	.827	-1.033	126	.304
	patient	22	.55	.963			
15 If you forget or omit your medication, do you forget it at noon?	physician	101	.43	.792	-1.310	121	.193
	patient	22	.68	.995			
16 If you forget or omit your medication, do you forget it in the evening?	physician	108	.64	.971	.136	129	.892
	patient	23	.61	.941			
17 Do you deliberately not take medications you do not consider important but take the rest?	physician	109	.17	.601	-.012	131	.991
	patient	24	.17	.482			
18 If you take medication as a syringe or a weekly tablet, have you ever forgotten it?	physician	105	.42	.731	1.052	127	.295
	patient	24	.25	.608			

Note. Displayed are results from unadjusted Student´s t-test. Results were confirmed by nonparametric group comparison (data not shown).
M = Mean, SD = standard deviation, df = degrees of freedom

Table S3. Binominal logistic regression: Medication changes initiated by patient or physician

Step		95% confidence interval						
		Exp(B)	Lower CI	Upper CI	R^2	χ^2	df	Sig.
1	Sex: Female	2.750	.731	10.341	.194	11.887	10	.293
	Age	1.092	1.005	1.186				
	Education level	.596	.115	3.093				
	Education level high	1.169	.235	5.808				
	Education level middle	1.082	.921	1.270				
	BDI	1.004	.915	1.102				
	HCCQ-D	.651	.364	1.161				
	MoCA	1.190	.882	1.607				
	Frequency of doctors appointments (quarterly)	.770	.462	1.284				
	SAMS total	.995	.889	1.113				
	Constant	.000						
2	Sex: Female	2.753	.733	10.341	.194	11.880	9	.220
	Age	1.092	1.005	1.185				
	Education level							
	Education level high	.591	.115	3.024				
	Education level middle	1.167	.235	5.793				
	Number of pills/day	1.084	.928	1.265				
	HCCQ-D	.644	.373	1.112				
	MoCA	1.187	.884	1.594				
	Frequency of doctors appointments (quarterly)	.767	.462	1.276				
	SAMS total	.995	.890	1.113				
	Constant	.000						
3	Sex: Female	2.775	.746	10.315	.194	11.872	8	.157
	Age	1.091	1.005	1.185				
	Education level							
	Education level high	.593	.116	3.032				
	Education level middle	1.175	.238	5.794				
	Number of pills/day	1.082	.929	1.262				
	HCCQ-D	.649	.384	1.097				
	MoCA	1.188	.886	1.595				
	Frequency of doctors appointments (quarterly)	.768	.462	1.276				
	Constant	.000						
4	Sex: Female	3.207	.916	11.223	.182	11.100	6	.085
	Age	1.077	1.000	1.160				
	Number of pills/day	1.085	.931	1.265				
	HCCQ-D	.664	.398	1.109				
	MoCA	1.166	.873	1.558				
	Frequency of doctors appointments (quarterly)	.794	.501	1.259				
	Constant	.000						
5	Sex: Female	3.055	.890	10.486	.165	10.025	5	.075
	Age	1.077	1.002	1.158				
	HCCQ-D	.676	.408	1.118				
	MoCA	1.114	.845	1.469				
	Frequency of doctors appointments (quarterly)	.823	.536	1.262				
	Constant	.000						

6	Sex: Female	2.847	.850	9.531	.156	9.436	4	.051
	Age	1.070	.996	1.148				
	HCCQ-D	.701	.430	1.142				
	Frequency of doctors appointments (quarterly)	.800	.522	1.227				
	Constant	.007						
7	Sex: Female	2.961	.895	9.802	.131	7.882	3	.049
	Age	1.064	.991	1.143				
	HCCQ-D	.712	.438	1.155				
	Constant	.006						
8	Sex: Female	2.912	.890	9.530	.102	6.066	2	.048
	Age	1.057	.987	1.132				
	Constant	.002						

Note: BDI = Beck's Depression Inventory II, HCCQ = Health Care Climate Questionnaire, MoCA = Montreal Cognitive Assessment, SAMS = Stendal Adherence to Medication Score

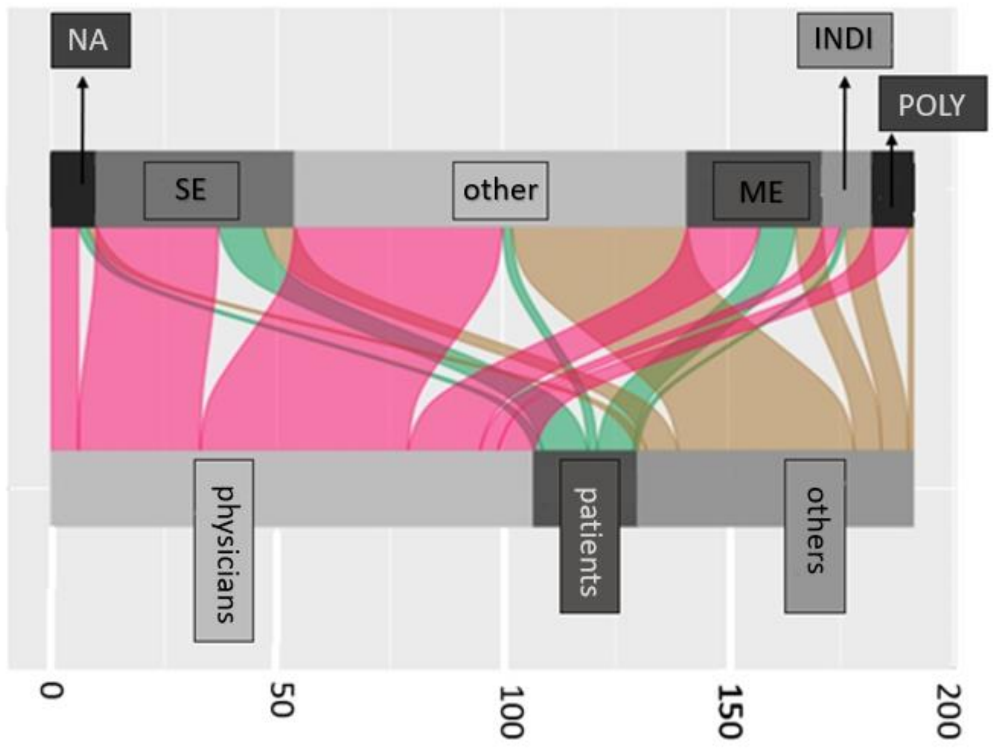

Figure S1. Alluvial plot for reasons of medication changes. note: NA = not available, INDI = new indication, ME = missing effects, SE = side effects, POLY = polymedication.

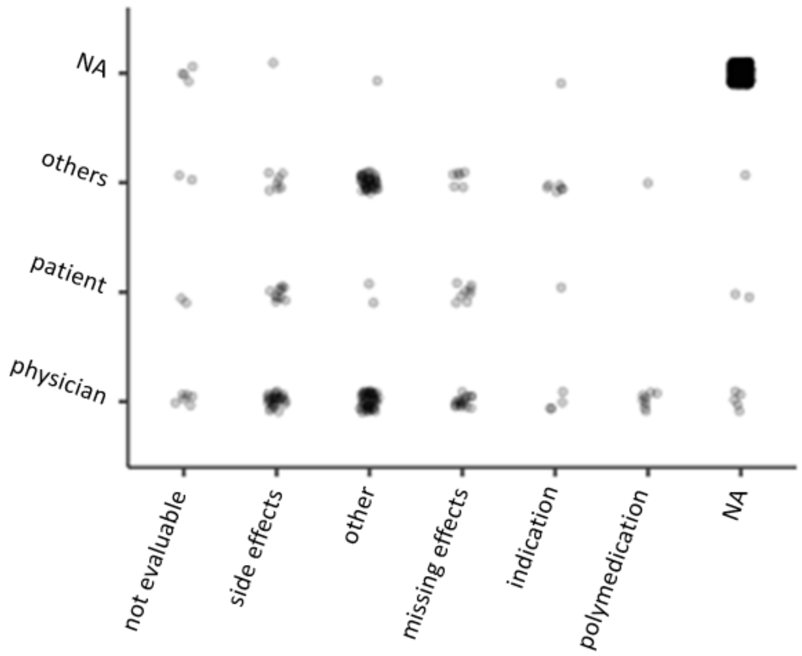

Figure S2. Scatter plot of bivariate relationship.
